# EEF2 Analysis Challenges the Monophyly of Archaeplastida and Chromalveolata

**DOI:** 10.1371/journal.pone.0002621

**Published:** 2008-07-09

**Authors:** Eunsoo Kim, Linda E. Graham

**Affiliations:** Department of Botany, University of Wisconsin-Madison, Madison, Wisconsin, United States of America; University of British Columbia, Canada

## Abstract

**Background:**

Classification of eukaryotes provides a fundamental phylogenetic framework for ecological, medical, and industrial research. In recent years eukaryotes have been classified into six major supergroups: Amoebozoa, Archaeplastida, Chromalveolata, Excavata, Opisthokonta, and Rhizaria. According to this supergroup classification, Archaeplastida and Chromalveolata each arose from a single plastid-generating endosymbiotic event involving a cyanobacterium (Archaeplastida) or red alga (Chromalveolata). Although the plastids within members of the Archaeplastida and Chromalveolata share some features, no nucleocytoplasmic synapomorphies supporting these supergroups are currently known.

**Methodology/Principal Findings:**

This study was designed to test the validity of the Archaeplastida and Chromalveolata through the analysis of nucleus-encoded eukaryotic translation elongation factor 2 (EEF2) and cytosolic heat-shock protein of 70 kDa (HSP70) sequences generated from the glaucophyte *Cyanophora paradoxa*, the cryptophytes *Goniomonas truncata* and *Guillardia theta*, the katablepharid *Leucocryptos marina*, the rhizarian *Thaumatomonas* sp. and the green alga *Mesostigma viride*. The HSP70 phylogeny was largely unresolved except for certain well-established groups. In contrast, EEF2 phylogeny recovered many well-established eukaryotic groups and, most interestingly, revealed a well-supported clade composed of cryptophytes, katablepharids, haptophytes, rhodophytes, and Viridiplantae (green algae and land plants). This clade is further supported by the presence of a two amino acid signature within EEF2, which appears to have arisen from amino acid replacement before the common origin of these eukaryotic groups.

**Conclusions/Significance:**

Our EEF2 analysis strongly refutes the monophyly of the Archaeplastida and the Chromalveolata, adding to a growing body of evidence that limits the utility of these supergroups. In view of EEF2 phylogeny and other morphological evidence, we discuss the possibility of an alternative eukaryotic supergroup.

## Introduction

Eukaryotes constitute one of the three domains of life, distinguished from bacteria and archaebacteria by their greater molecular, cellular, and reproductive complexity. About 1.5 million species of eukaryotes have been recognized and named thus far, with at least several times that number remaining to be catalogued [1]. Much of eukaryotic diversity occurs among protists, whose high-level classification remains uncertain in spite of the need for a reliable, phylogeny-based classification in ecological, medical, and industrial research.

Eukaryotes can be conservatively classified into about 60 robust lineages based primarily on ultrastructural features [2], [3]. Alternatively, eukaryotes have been grouped into only two major clades—unikonts and bikonts–based largely on a single gene fusion event, under the assumption that parallel fusions would be improbable [4]–[6]. However, this assumption is refuted by evidence that gene fusion events do occur independently in different eukaryotic groups [7]. A fundamental unikont-bikont dichotomy is also questioned by the phylogenetic position of the bikont Apusozoa among the “unikonts”, as well as other data [8], [9]. Other recent authors have classified eukaryotes into 5 or 6 major supergroups: Amoebozoa, Opisthokonta, Archaeplastida (or Plantae), Chromalveolata, Rhizaria, and Excavata, with the first two grouped as ‘unikonts’ by some authors [10]–[12]. However, the validity of some of these supergroups, notably Excavata, Archaeplastida and Chromalveolata, is controversial [13]–[21].

The present study was designed to test monophyly of the Archaeplastida and Chromalveolata, each defined by a single primary- or secondary-plastid generating endosymbiotic event [10]. The Archaeplastida is composed of three well characterized monophyletic groups: the Glaucophyta, Rhodophyta (i.e., red algae), and Viridiplantae (i.e., green algae plus land plants) [10]. All members of the Archaeplastida possess double membrane-bound plastids (i.e., primary plastids), which are believed to have been derived directly from a cyanobacterial endosymbiont by primary endosymbiosis [22]. It should be noted that the rhizarian *Paulinella chromatophora* (which does not group with the Archaeplastida) independently acquired photosynthetic bodies directly from a cyanobacterium [23], [24], although there is debate whether to designate these entities as ‘plastids’ or ‘endosymbionts’ [25]–[27]. The Chromalveolata comprises four monophyletic groups—Alveolata, Cryptophyta (plus Katablepharidae) [8], [28], Haptophyta, and Stramenopiles, each group containing at least some members harboring plastids thought to be derived from a red alga by secondary endosymbiosis [10]. Cryptophytes, haptophytes, and stramenopiles were grouped as ‘chromists,’ based on shared features of their plastids. These shared features include the presence of four-bounding membranes and, with some exceptions [29], confluence of the outermost plastid membrane with the nuclear envelope [30]. It should to be noted, however, that several “early-diverging” clades within the stramenopiles, and the cryptophyte genus *Goniomonas* (plus katablepharid species), do not possess plastids. Alveolates include ciliates (plastid-less), apicomplexans, and dinoflagellates, the latter including many plastid-less as well as plastid-bearing members [31]. Most plastid-bearing dinoflagellates have peridinin as a major carotenoid fraction and their plastids are generally enclosed by three membranes [32], whereas plastids that are present in the majority of apicomplexans (i.e., apicoplasts) are exclusively non-photosynthetic and are bound by 2–4 membranes [33]–[36]. Recently, a photosynthetic relative of the apicomplexans has been identified which harbors plastids with four membranes that are related to apicoplasts [37]. In contrast to most ‘chromist’ plastids, the outermost plastid membranes of alveolate plastids are not connected to the nuclear membrane [32], [35].

The major issues surrounding the endosymbiotic origins of plastid-bearing eukaryotes can be summarized by the following questions: a) Did the plastids of the Archaeplastida taxa arise from a single or multiple source(s) of cyanobacteria [38]–[40]? ; b) Are all these plastids derived from a single endosymbiotic event [18], [41]? ; c) Can the plastids of the Chromalveolata taxa be traced back to a single red algal type [42]? ; d) Were chromalveolate plastids acquired once or on multiple occasions [43], [44]?

As a means of addressing some of these evolutionary concerns, we carefully targeted molecular phylogenetic markers and taxa to the specific issue of the monophyly of the Archaeplastida and Chromalveolata. More focused analyses such as ours can reveal strong gene-specific evidence for or against phylogenetic relationships, which might be overlooked or unrecognizable in concatenation analyses [45]. We chose two conserved, nuclear protein-coding genes that have been widely used to evaluate eukaryotic diversification: EEF2 (eukaryotic translation elongation factor 2) and cytosolic HSP70 (heat-shock protein of 70 kDa) genes. We generated sequence data from representatives of major eukaryotic phyla, including cryptophytes, a glaucophyte, a green alga, a katablepharid, and a rhizarian and analyzed these new sequences together with existing available database sequences for other eukaryotic taxa.

HSP70 is a molecular chaperone which assists in assembly and folding of proteins and occurs universally in all organisms [46]. Because of its highly conserved sequence, HSP70 has been extensively surveyed to address some of the most ancient evolutionary events such as early bacterial, archaebacterial, and eukaryotic diversification patterns [46], [47]. Like HSP70, EEF2 (and its prokaryotic homolog EF-G) is also highly conserved. EEF2 constitutes an essential component of the translational machinery, where it is involved in the protein elongation step, specifically in the translocation of tRNAs and mRNA [48], [49]. EEF2 is valued as a phylogenetic marker because of its large size (∼800 amino acids) and consequently its potential to retain more phylogenetic signal than smaller proteins. In a previous study, EEF2 phylogeny strongly suggested a sister relationship between rhodophytes and Viridiplantae; this observation was argued as nucleocytoplasmic evidence in support of a single endosymbiotic origin for primary plastids [50]. Although EEF2 sequences from glaucophytes could critically test Archaeplastida monophyly, such sequences are so far not available.

In this study, we determined six EEF2 and four cytosolic HSP70 sequences from diverse eukaryotic groups. Importantly, our EEF2 and cytosolic HSP70 phylogenies included, for the first time, nearly full-length sequences of glaucophytes, katablepharids, or cryptophytes. The results of our study, specifically the EEF2 analysis, strongly refute the monophyly of the Archaeplastida and Chromalveolata.

## Results

### EEF2 and cytosolic HSP70 analyses

**Figure 1:**
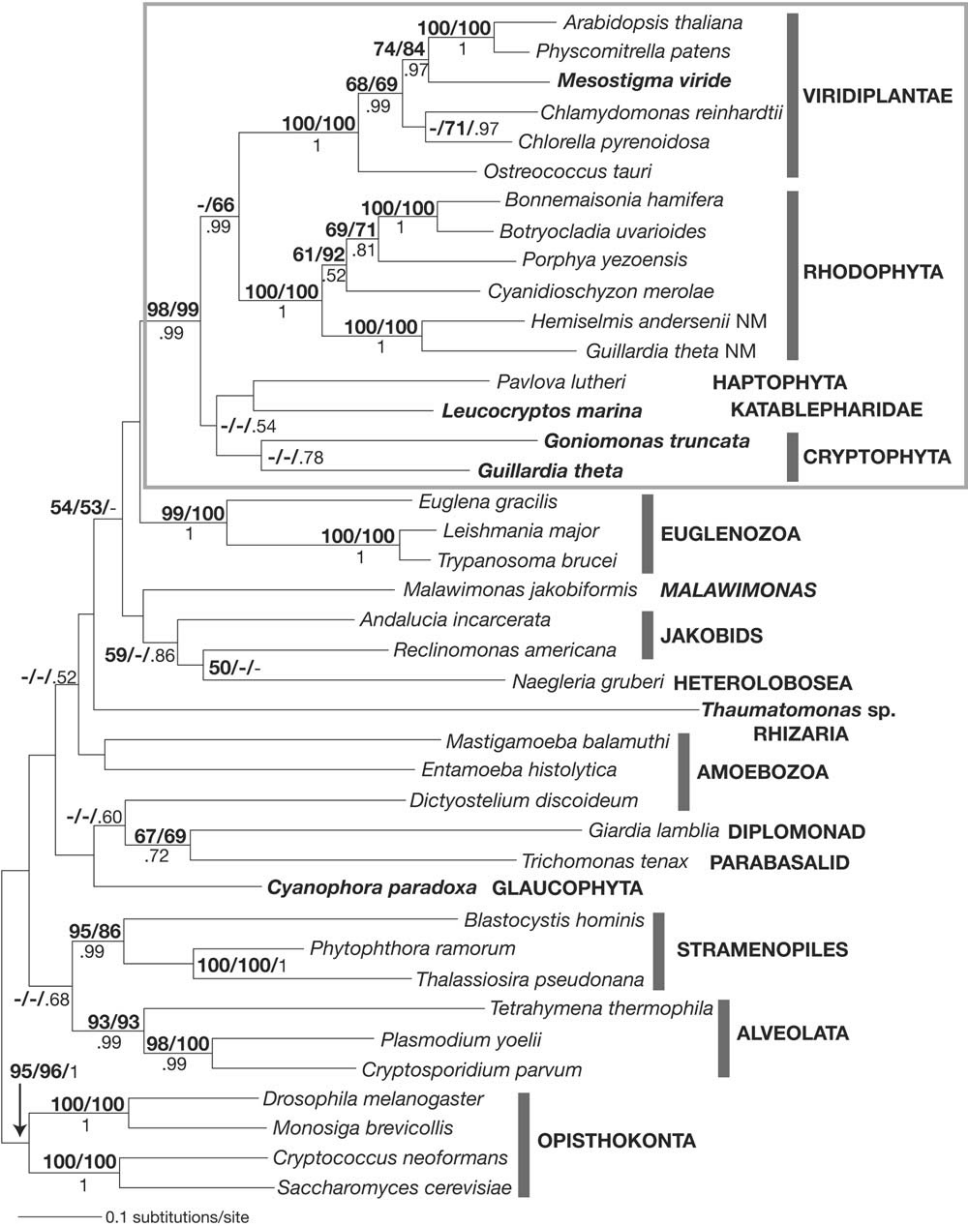
Maximum likelihood tree based on the EEF2 alignment, under the WAG+Γ+I+F model of protein evolution (RAxML). Bootstrap support values >50% (RAxML/FastME) and posterior probabilities >0.50 are indicated at the corresponding nodes. Sequences newly obtained in this study are labeled in bold. Note that the Viridiplantae, Rhodophyta, Haptophyta, Katablepharidae, and Cryptophyta formed a well-supported clade. NM stands for nucleomorph.

EEF2 phylogenetic trees inferred from maximum likelihood (RAxML, PhyloBayes) and distance (FastME) methods were more or less similar, although some deep branching patterns that had low bootstrap support, differed (data not shown). In some cases posterior probabilities (PhyloBayes) were very high (>0.95) even when respective bootstrap values (RAxML, FastME) were low (<70%), although these numbers are not directly comparable as they have different statistical interpretations. For this reason, we interpreted a given relationship as being well supported only when all three supporting values (bootstrap and posterior probability) were high (>90% or >0.90). In all three analyses, a number of well-established eukaryotic groups, including rhodophytes, alveolates, opisthokonts, euglenozoans, and Viridiplantae were recovered with >90% bootstrap support and high posterior probabilities (Figure 1). Most interestingly, unlike what is expected from the Archaeplastida proposal, the glaucophyte *C. paradoxa* did not branch with rhodophytes or Viridiplantae in the EEF2 tree. Instead, EEF2 analysis identified a well-supported clade composed of cryptophytes, katablepharids, haptophytes, rhodophytes, and Viridiplantae (Figure 1). Within this large clade, relationships among the five eukaryotic groups were poorly resolved. Although Viridiplantae and rhodophytes were each other's closest sister group, bootstrap values supporting this relationship were low (49% for RAxML and 66% for FastME) (Figure 1).

**Figure 2:**
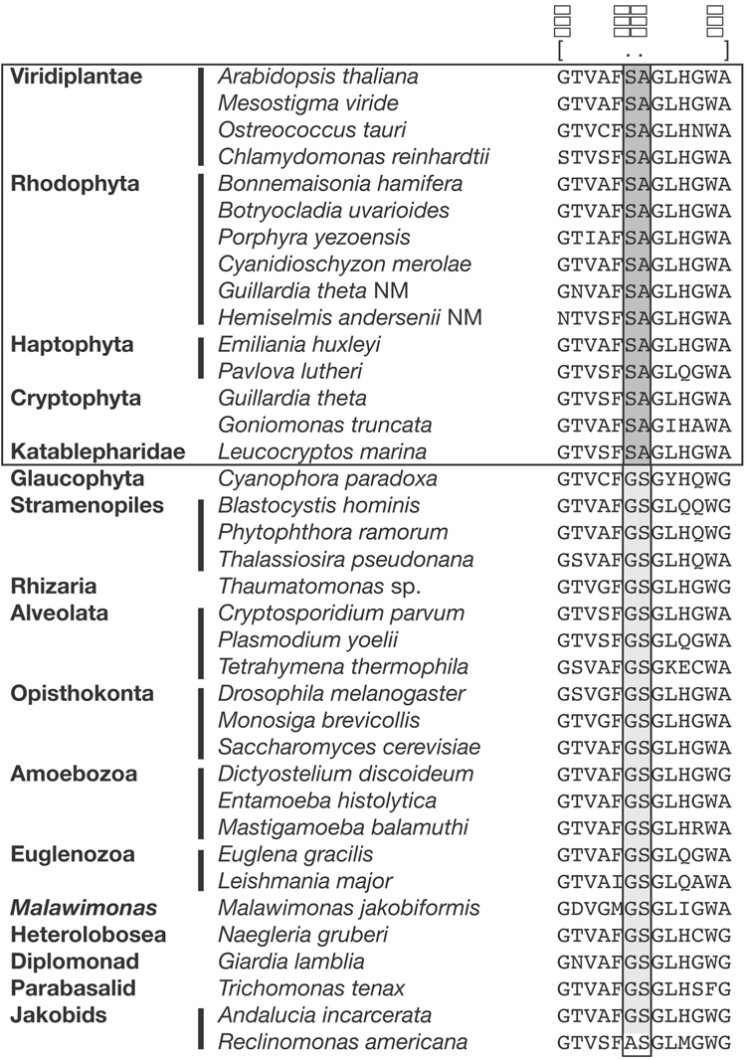
Two amino acid signature within the EEF2. Note that EEF2 of Viridiplantae, Rhodophyta, Haptophyta, Katablepharidae, and Cryptophyta have amino acids serine and alanine at positions 212 and 213, whereas most other eukaryotes have glycine and serine residues instead.

Support for the monophyly of the EEF2 of cryptophytes, katablepharids, haptophytes, rhodophytes, and Viridiplantae is further provided in the form of a two amino acid signature (Figure 2). In these EEF2, two consecutive amino acids, serine (S) followed by alanine (A), occur at positions 212 and 213 whereas most other taxa encode the highly conserved amino acid sequences, glycine (G) and serine (S), at these positions (Figure 2). This suggests that the SA amino acids arose via amino acid replacement of the ancestral GS residues.

In contrast to EEF2, major eukaryotic relationships were largely unresolved in the cytosolic HSP70 phylogeny (Figure S1). Of well-established eukaryotic groups, only the alveolates, stramenopiles, euglenozoans, and rhizaria were recovered with >50% bootstrap support and monophyletic cryptophytes, opisthokonts, rhodophytes, and Viridiplantae were not recovered in the ML tree (Figure S1). As most nodes in the ML tree were poorly supported, it is not clear whether some abnormal branching patterns in the cytosolic HSP70 tree are simply due to the lack of informative sites or other factors (e.g., incomplete lineage sorting, horizontal gene transfer, paralogy) that can lead to discordance between the gene and the species trees. HSP70 phylogeny is also known to be susceptible to the long branch attraction (LBA) artifact [51].

### Concatenated protein phylogeny

**Figure 3:**
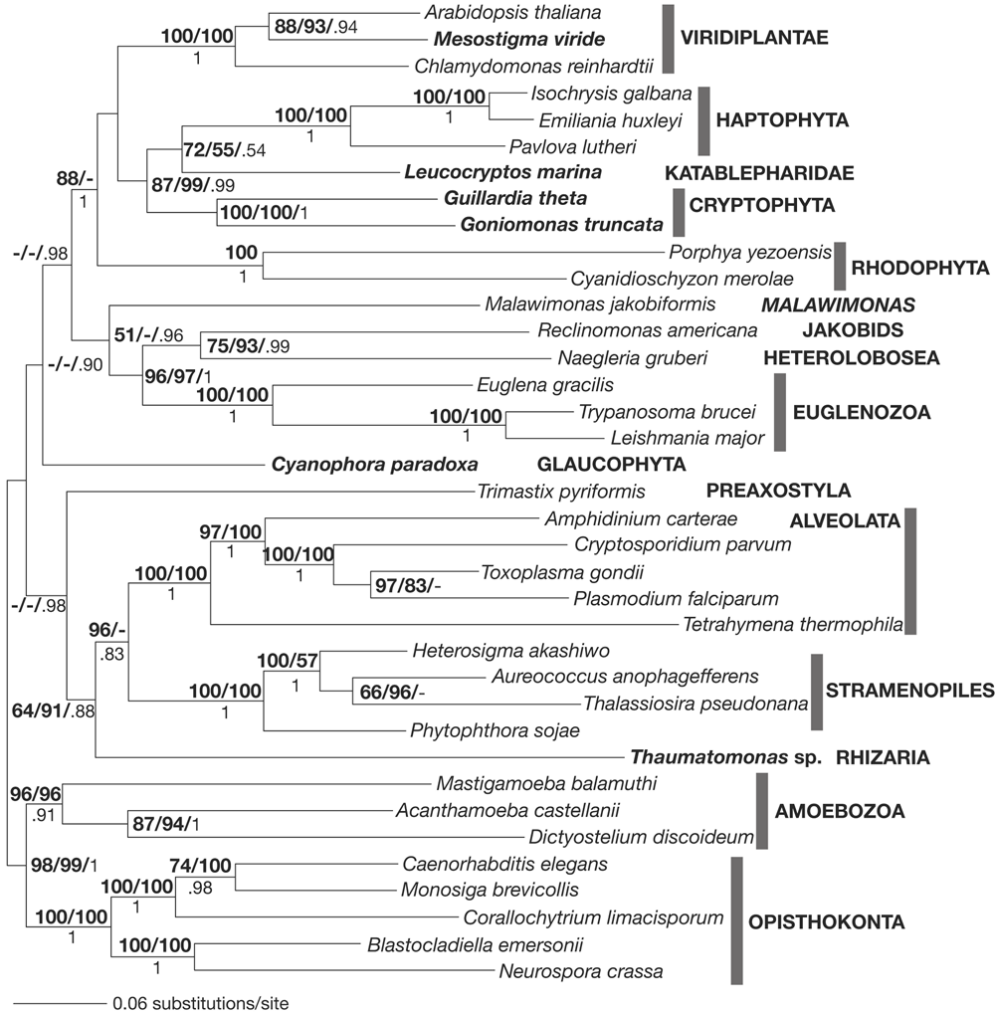
Maximum likelihood tree for the concatenated protein data set, under the WAG+Γ+I model of protein evolution (RAxML) with the unlinked option. Included proteins are EEF2, actin, cytosolic HSP70, cytosolic HSP90, and α-tubulin, and β-tubulin. Bootstrap support values >50% (RAxML/FastME) and posterior probabilities >0.50 are shown.

As single-gene trees are generally poorly resolved due to the presence of limited phylogenetic signal [52], a combined analysis of six proteins—α-tubulin, β-tubulin, actin, cytosolic HSP70, cytosolic HSP90, and EEF2—was performed in an attempt to improve the resolution of the tree by increasing the number of informative characters. Up to about 40% of missing data for a particular taxon was permitted for increasing taxonomic sampling, important for the accuracy of phylogenetic inference [52]–[54]. The final alignment included 2,797 amino acids with 278 constant sites and had in total 5.23% missing data. Well-established groups including the alveolates, cryptophytes, euglenozoa, haptophytes, rhodophytes, opisthokonts, stramenopiles, and Viridiplantae were recovered with strong bootstrap support and >0.95 posterior probabilities (Figure 3). In addition, higher level-groupings such as the Opisthokonta-Amoebozoa and the Euglenozoa-Heterolobosea clades received strong support (Figure 3). Cryptophyta, Katablepharidae, and Haptophyta formed a clade with moderate to strong support, which is consistent with recent multiple-gene phylogenies that suggested a close relationship between Cryptophyta and Haptophyta (Katablepharidae was not examined in these studies) [55], [56]. As in the EEF2 analysis, a clade comprising the Cryptophyta, Katablepharidae, Haptophyta, Rhodophyta, and Viridiplantae was recovered in the combined protein tree. Although the clade received the highest posterior probability of 1.0 in both analyses, bootstrap support values for the clade decreased from 98 or 99% in the EEF2 tree to 88 or <50% in the combined analysis (compare Figures 1 and 3). The glaucophyte *C. paradoxa* did not branch close to rhodophytes or Viridiplantae and its phylogenetic position to other eukaryotic groups was unresolved. Lastly, the rhizarian *Thaumatomonas* sp. branched with alveolates and stramenopiles with weak to moderate support values, consistent with a previous study based upon >100 concatenated protein sequences [14].

## Discussion

### EEF2 phylogeny refutes the monophyly of Archaeplastida and Chromalveolata

Moreira et al. [50] showed in their EEF2 tree that rhodophytes and Viridiplantae were closely related to each other and that a sister relationship was strongly supported, with a 100% ML bootstrap value. Prior to that, the hypothesis that a single plastid-generating endosymbiotic event occurred at the origin of glaucophytes, rhodophytes and Viridiplantae was primarily, if not entirely, based on plastid-related features, because no nucleocytoplasmic data in support of the hypothesis were available. Therefore, the Moreira et al. EEF2 result was regarded as the first strong nucleocytoplasmic evidence favoring a monophyletic origin of the Archaeplastida [18], [57], although glaucophytes were not examined in their study. Subsequently, an analysis based on >100 concatenated nucleus-encoded proteins indicated that glaucophytes branched closely to rhodophytes and Viridiplantae [58]. Together with plastid-related evidence (see below for details), these results have convinced many researchers in the field that the controversy surrounding the origin of primary plastids was settled (e.g. [11]). However, both the Moreira et al. [50] and Rodriguez-Ezpeleta et al. [58] studies suffered from inadequate taxonomic sampling, notably lacking sequences of cryptophytes, katablepharids, and haptophytes, which appear to be critical in evaluating the validity of the supergroup Archaeplastida as well as the Chromalveolata (see discussion below). In our EEF2 analysis, which included glaucophytes, cryptophytes, and katablepharids, the monophyly of the Archaeplastida and Chromalveolata was strongly refuted. In addition, the specific affiliation of rhodophytes and Viridiplantae is no longer significantly supported, although they still form a well-supported clade together with cryptophytes, katablepharids, and haptophytes (Figure 1).

In recent studies, the strong associations among haptophytes, rhodophytes, and Viridiplantae in EEF2 phylogenies were interpreted as evidence for lateral gene transfer from a red algal endosymbiont to the haptophyte nucleus [56], [59]. However, with the addition of our new cryptophyte and katablepharid EEF2 sequences, it is now clear that the earlier proposal is no longer tenable (Figure 1). Cryptophytes have a copy of EEF2 gene in the nucleomorph genome, in addition to one or more nucleus-encoded copies [60], [61]. In our study, as is predicted from its red algal ancestry, the cryptophyte nucleomorph-encoded EEF2 branched close to the red algal EEF2 (Figure 1). In contrast, the nucleus-encoded EEF2 of cryptophytes, katablepharids, and haptophytes did not show specific affiliation to the nucleomorph or red algal copies (Figure 1). These branching patterns suggest that the EEF2 gene residing in the nuclei of haptophytes, cryptophytes, and katablepharids was not obtained through endosymbiotic gene transfer from the red algal endosymbiont and probably descended vertically from their ancestors. In addition, the hypothesis of an endosymbiotic gene transfer of EEF2 gene requires the *a priori* assumption that katablepharids and the cryptophyte genus *Goniomonas* once possessed plastids, although there is no molecular or ultrastructural evidence of plastids in these lineages [62], [63].

### Concatenated protein analysis

Neither the monophyly of the Archaeplastida nor the Chromalveolata were recovered in our concatenated six protein phylogeny (Figure 3). It should be noted, however, that the clade comprising cryptophytes, katablepharids, haptophytes, rhodophytes, and Viridiplantae is no longer significantly supported by bootstrap values. One possible reason might be a long-branch effect of red algal-derived sequences, especially as their actin and β-tubulin sequences are relatively quite divergent (data not shown). On the other hand, it cannot be completely ruled out that compared to other molecular markers, EEF2 has disconcordant phylogenetic signal, although EEF2 phylogeny does not show any obvious signs of conflict with the five other protein markers examined in this study (data not shown). Nevertheless, it is difficult to differentiate between these two possibilities given the fact that not many other nucleus-encoded molecular markers have been examined at a similar level of taxonomic sampling. Finally, it is worth mentioning that in a study of >100 concatenated nucleus-encoded protein sequences (albeit with more than 50% of sequence data missing for cryptophytes and haptophytes), the phylogenetic relationships of cryptophytes & haptophytes, rhodophytes, or Viridiplantae to other eukaryotic groups remained unresolved [55]. This suggests that use of markers selected specifically for their information value may be an effective alternative to inferring deep phylogenies by the concatenation approach (or total evidence approach). Given that individual molecular markers can have differing histories due to lateral gene transfer, hidden paralogy, and deep coalescence [64], a concatenation approach can potentially hide strong local phylogenetic signal.

### Evaluation of the supergroup Archaeplastida

Over the years, the origin of the plastids in glaucophytes, rhodophytes and Viridiplantae has drawn considerable attention [18], [19], [38], [40], [65]. These plastids are known as primary plastids as they are thought to have arisen directly from a cyanobacterial ancestor that was engulfed by an eukaryotic host. Several molecular and genomic data support the notion that these primary plastids arose from a single cyanobacterial endosymbiont. Two particularly compelling pieces of evidence supporting this hypothesis are the presence of an inner plastid membrane translocon Tic110 protein [40], [66] and a unique *atpA* gene cluster [67]. These features are common to the plastids of the Viridiplantae, glaucophytes, and rhodophytes, but not found in the cyanobacteria examined thus far. These features may represent post-endosymbiotic inventions that occurred prior to the diversification of the three ‘primary’ plastids [40], [67], although it is also possible that they may have been characteristic of ancestral cyanobacteria of a type so far undiscovered among modern taxa. Triple-helix chlorophyll-binding, light-harvesting antenna complexes (LHCs) have been suggested as another case of post-endosymbiotic innovation [68]. Because LHC homologs have not been identified in glaucophytes [69], such LHCs may have evolved after divergence of the glaucophyte plastid. Plastid genome content and gene phylogenies suggest a single origin of glaucophyte, rhodophyte and Viridiplantae plastids [58], [70], although such results do not completely rule out alternative hypotheses [38]. We also note that some other features once considered specific to plastids, such as inverted repeats in rRNA and the *psbB* gene cluster [40], [67], [71] have been subsequently identified in cyanobacteria, and thus no longer support (nor refute) a single origin hypothesis [39]. In summary, although the hypothesis of a common ancestry for red, green, and glaucophyte plastids is best supported by current data, additional genomic and molecular data for cyanobacteria are needed to further test the hypothesis.

In contrast to their plastids, little or no evidence supports an hypothesis of a common ancestry for the host (i.e., nucleocytoplasmic) component of Viridiplantae, glaucophytes, and rhodophytes. These three lineages differ in ultrastructure and biochemistry [72]. In addition, nucleus-encoded gene phylogenies have often been inconclusive [13], [19], [73], [74]. Although in large-scale phylogenies based on concatenated databases the monophyly of Viridiplantae, glaucophytes, and rhodophytes was initially recovered with strong support [58], the addition of cryptophyte or haptophyte sequences significantly lowered or eliminated support for monophyly of the three lineages [14], [55]. Likewise, mitochondrion-encoded gene phylogenies remain largely inconclusive as to the relationship between Viridiplantae and red algae [75]–[77] (mitochondrial genome data for the glaucophyte taxa are not publicly available for analysis).

Furthermore, although the mechanism of plastid origin by primary endosymbiosis is widely accepted [78], this concept is primarily based on the presence of two plastid membranes, which may not be a reliable marker if membranes have been lost over time [41]. Some dinoflagellates, for example, have plastids with two membranes that clearly are not of primary origin [32]. Another example is provided by the transient plastids (i.e., kleptoplastids) of the sea slug *Elysia chlorotica*, which have only two membranes, despite their origin from the stramenopiles *Vaucheria litorea*. Such kleptoplastids apparently lost two of the four original plastid membranes [79]. These observations suggest that loss of plastid membranes can occur during or after the engulfment of algal endosymbionts, potentially masking secondary or tertiary origin.

In summary, current data do not provide strong evidence for monophyly of the host lineage of the Viridiplantae, glaucophytes, and rhodophytes, whereas our EEF2 data strongly refute the concept of the Archaeplastida. The observed discrepancy between the nucleocytoplasmic and the plastid genealogy might be better explained by postulating multiple acquisitions of plastids in these eukaryotic lineages. If so, at least one of the ‘primary’ plastids may actually be of secondary origin.

### Evaluation of the supergroup Chromalveolata

The chromalveolate hypothesis, namely that cryptophytes, haptophytes, stramenopiles, and alveolates arose from a common ancestor via a secondary endosymbotic event [30], continues to be debated [15], [17], [20], [43]. The presence of many, early-diverging plastid-less taxa within stramenopiles and alveolates [80]–[82], and accumulating molecular data, generally conflict with the chromalveolate hypothesis or require massive plastid losses, despite the value of plastids in amino acid, fatty acid and heme biosynthesis, as well as photosynthesis [13], [15]. Lack of any sort of evidence from nucleus-encoded gene phylogenies casts further doubt on the chromalveolate hypothesis [8], [13]. Although the nucleus-encoded, plastid targeted glyceraldehyde-3-phosphate dehydrogenase (GAPDH) phylogeny has been presented as evidence for the chromalveolate hypothesis, cytosolic GAPDH sequences among ‘chromalveolate’ taxa did not form a clade, indicating that homologs have discordant evolutionary histories [44], [83]. In addition, the plastid-targeted GAPDH tree [84] is inconsistent with accepted organismal relationships; the apicomplexan *Toxoplasma gondii* is a sister to haptophytes with strong support, to the exclusion of peridinin-type dinoflagellates [85]. Overall, the GAPDH phylogenies seem to be more consistent with multiple occasions of plastid acquisition among ‘chromalveolate’ taxa. Plastid-encoded gene phylogenies vary in their level of support for the chromalveolate hypothesis, depending on taxonomic sampling and types and number of analyzed genes [42], [86]–[88]. Even when monophyly of the ‘chromalveolate’ plastids is recovered, it is also consistent with the “serial hypothesis”, which postulates serial transfer of red algal-derived plastids among ‘chromalveolates’ [88], [89]. Finally, recent molecular phylogenies showing that rhizaria are closely related to stramenopiles and alveolates [14], [56], together with EEF2 evidence presented here appear to deal a fatal blow to the chromalveolate hypothesis.

### Evidence for a new eukaryotic supergroup

Based on EEF2 and some morphological data, we propose an alternative eukaryotic supergroup that includes cryptophytes, katablepharids, haptophytes, rhodophytes, and Viridiplantae. We suggest the name Plastidophila (“friendly to plastid”) for the potential clade, because most subclades, except for katablepharid species and one cryptophyte genus (*Goniomonas*), are dominated by plastid-bearing members. Although genomic evidence for Plastidophila is yet limited, some morphological features shared among katablepharids, cryptophytes and Viridiplantae, especially “early-diverging” prasinophyte green algae, are consistent with this new concept [62], [90]–[92]. For instance, ejectisomes (i.e., ejectile organelles) of katablepharids are similar to those of the prasinophyte green alga *Pyramimonas*. Although ejectisomes of *Pyramimonas* form as a spirally coiled ribbon and those of katablepharids take the shape of an elongated tube with a single straight slit, both types discharge into a linear structure [90]. Further, two central flagellar microtubules that do not penetrate into the flagellar insertion area occur in both prasinophytes and katablepharids-cryptophytes [91]. In addition, both the katablepharid *Kathablepharis ovalis* and the prasinophyte *Pyramimonas* possess electron-dense material below the flagellar terminal plate [91]. The striated root that occurs in katablepharids has been suggested to be homologous to the system I fibrous roots found in Viridiplantae [91]. Finally, cell surfaces consisting of a basal fibrous layer and an upper scaly layer is common to katablepharids [90] and scaly green algae such as the “basal” streptophyte green alga *Mesostigma* [93] and the prasinophyte *Tetraselmis* [94]. Based on comparative morphology, Lee and Kugrens [90] and Lee et al. [91] suggested that katablepharids represent evolutionary intermediates between cryptophytes and Viridiplantae. A close relationship between katablepharids and cryptophytes is supported by SSU and LSU rRNA phylogenies [8], [28]. Recent analyses based on concatenated protein data sets also suggest a sister relationship between the haptophytes and cryptophytes (katablepharids were unexamined), although the phylogenetic position of this clade relative to other eukaryotes remained unresolved [55], [56]. Consistent with these results, our analyses also suggested that cryptophytes, katablepharids, and haptophytes are closely related to each other, although a specific relationship between cryptophytes and katablepharids was not recovered. If the cryptophyte-katablepharids-haptophyte clade and the Plastidophila supergroup suggested by EEF2 phylogeny are indeed correct, it follows that morphological traits common to katablepharids and “early-diverging” green algae might represent features that were shared by the common ancestor of Plastidophila. Hence, katablepharids (plus the cryptophyte *Goniomonas*) may be useful models of the heterotrophic flagellate that was ancestral to the photosynthetic lineage that led to land plants and other algae within the Plastidophila. Genomic analysis of katablepharids and the cryptophyte *Goniomonas* may illuminate nucleocytoplasmic traits of the plant lineage that existed prior to the massive invasion of genes from a cyanobacterial precursor to the plastid [86].

### Conclusion

The concepts of Archaeplastida and Chromalveolata do provide a simple way to explain the distribution of primary and secondary plastids by minimizing the number of plastid-generating endosymbiotic events required. However, our EEF2 data add to a growing body of evidence that refutes the Archaeplastida and Chromalveolata. By fostering inaccurate assumptions of relationships, continued use of these supergroup concepts may be deleterious to progress in studies of ecologically, medically, and industrially important protists. Given the lack of support for the monophyly of the Archaeplastida and Chromalveolata, it is sensible to consider alternative evolutionary models. Based on EEF2 analysis and some ultrastructural traits, we suggest testing the concept of a supergroup Plastidophila that links katablepharids-cryptophytes, haptophytes, rhodophytes, and Viridiplantae.

## Materials and Methods

### Sequencing of EEF2 and cytosolic HSP70 genes

Genomic DNA and/or cDNA were purified from *Cyanophora paradoxa*, *Goniomonas truncata*, *Leucocryptos marina* (NIES 1335), *Mesostigma viride*, and *Thaumatomonas* sp. as described in Kim et al. (2006). EEF2 and cytosolic HSP70 genes, typically ∼2.5 Kbp and ∼2.0 Kbp in size excluding intron regions, are considered relatively large for PCR amplification protocols that employ degenerate primers, so consequently, 2–3 overlapping fragments were PCR amplified and sequenced to obtain nearly full-length sequences of each gene or cDNA. Degenerate primers of about 20–30 bp in size were designed to target conserved sequence regions across diverse eukaryotic taxa within EEF2 and cytosolic HSP70 genes (Table S1). In most cases, the use of these degenerate primer pairs enabled the amplification of only partial regions, hence species-specific primers were subsequently identified from partial sequencing and used to amplify the adjacent fragment(s) (Table S2). EST data for *C. paradoxa* and *M. viride* were utilized to identify species-specific primer sites for EEF2 gene amplifications of these organisms. In many cases, a two-step nested PCR approach was adapted to obtain larger amounts of PCR fragments from very little starting DNA material. PCR amplification, PCR fragment cloning, and sequencing were performed as previously described [8]. As eukaryotes encode 3 or 4 types of HSP70 (i.e., cytosolic, ER, mitochondrial, and plastid forms), each sequenced HSP70 fragment was carefully examined to verify that it contained signature sequence sites for the cytosolic form [95], [96]. The EEF2 sequence of *G. theta* was retrieved from the 4× genome assembly, generated by the US Department of Energy Joint Genome Institute (http://www.jgi.doe.gov/). Newly obtained EEF2 and cytosolic HSP70 sequences were deposited in GenBank with accession numbers EU812174–812204 (Table S2).

### Molecular sequence analysis

Newly obtained EEF2 and HSP70 sequences were manually assembled and aligned to sequences downloaded from GenBank using MacClade ver. 4.08 [97]. Ambiguous regions were excluded. Phylogenetic analysis was performed based on deduced amino acid sequences to minimize phylogenetic artifacts caused by codon usage variations [98]. The final EEF2 and cytosolic HSP70 sequence alignments included 736 and 462 amino acid sites and had 1.08% and 1.28% missing data, respectively. The two alignments were analyzed individually and were combined with α-tubulin, β-tubulin, actin, and cytosolic HSP90 alignments [8] for concatenated protein analysis.

Maximum likelihood analysis of amino acid sequence alignments was performed using RAxML ver. 7.0.4 [99] and PhyloBayes ver. 2.3 [100]. For RAxML analysis, ML trees were inferred with the WAG+Γ+I+F for the EEF2 data and the WAG+Γ+I for the concatenated protein data (4 discrete gamma rates), and from 100 distinct randomized maximum parsimony starting trees. The models of protein evolution were selected using ProtTest ver. 1.4 [101]. For the concatenated data set, the ‘-M’ option was applied so that each protein partition had its own branch length. Bootstrap analysis was based on 100 re-samplings. For analysis with PhyloBayes, constant sites were deleted and the CAT+Γ model of protein evolution with 4 discrete categories for gamma distributed rates was applied [100]. Markov chains were run for 60,000 cycles, the first 5,000 points were discarded as burn-in, and every 10^th^ tree from the remaining points was collected to compute the posterior probabilities for individual nodes. For each analysis, two chains were run in parallel and compared to check for convergence.

Protein distance analysis was performed using TREE-PUZZLE ver. 5.2 [102] and FastME [103]. For TREE-PUZZLE analysis, pairwise maximum likelihood distances were estimated under the WAG+Γ+I model with 4 and 8 discrete Gamma distribution rates for EEF2 and the concatenated data set, respectively. The resulting distance matrices were then used to construct distance trees using FastME with the initial tree construction option of the Greedy Minimum Evolution algorithm and the tree swapping option of the Balanced Nearest Neighbor Interchanges algorithm. Bootstrap analysis was based on 100 re-samplings using puzzleboot ver. 1.03 (available from www.tree-puzzle.de). Bootstrap datasets were generated using the SEQBOOT program from the PHYLIP package ver. 3.66 [104].

## Supporting Information

Figure S1 (0.37 MB EPS)

Table S1 (0.27 MB EPS)

Table S2 (0.28 MB EPS)
